# [Expression of Concern] CDKN3 is an independent prognostic factor and promotes ovarian carcinoma cell proliferation in ovarian cancer

**DOI:** 10.3892/or.2025.9008

**Published:** 2025-10-16

**Authors:** Tianren Li, Hui Xue, Yi Guo, Kejun Guo

Oncol Rep 31: 1825–1831, 2014; DOI: 10.3892/or.2014.3045

Following the publication of this paper, it was drawn to the Editor's attention by a concerned reader that the cell invasion assay data shown in the three panels of Fig. 6A appeared to contain overlapping sections at different orientations (all three panels), even though the data were apparently meant to show the results of experiments performed under different conditions.

The authors were contacted by the Editorial Office to offer an explanation for this apparent anomaly in the presentation of the data in this paper; however, up to this time, no response from them has been forthcoming. Owing to the fact that the Editorial Office has been made aware of potential issues surrounding the scientific integrity of this paper, we are issuing an Expression of Concern to notify readers of this potential problem while the Editorial Office continues to investigate this matter further.

